# Photodynamic Activation of Mammalian and Avian Cholecystokinin Type 1 Receptor Outside of the Pancreatic Acinar Cell Microenvironment

**DOI:** 10.3390/ijms262412011

**Published:** 2025-12-13

**Authors:** Jie Wang, Zong Jie Cui

**Affiliations:** 1 Department of Biology, College of Life Sciences, Beijing Normal University, Beijing 100875, China; 2 The Ministry of Education Laboratory for Cell Proliferation and Regulation, College of Life Sciences, Beijing Normal University, Beijing 100875, China

**Keywords:** G protein coupled receptor (GPCR), photodynamic biology (PDB), reactive oxygen species (ROS), redox signaling, cholecystokinin type 1 receptor, singlet oxygen (^1^O_2_), calcium oscillations, miniSOG, sulfonated aluminum phthalocyanine (SALPC)

## Abstract

Cholecystokinin 1 receptor (CCK1R) is activated by singlet oxygen (^1^O_2_) in type II photodynamic action in isolated rat, mouse, and Peking duck pancreatic acini. To examine whether this is maintained outside the microenvironment of pancreatic acinar cell, photodynamic activation of CCK1R from human, rat, mouse, and Peking duck expressed in CHO-K1 cells was examined, as monitored with Fura-2 fluorescence calcium imaging. Photodynamic action with sulphonated aluminum phthalocyanine was found to trigger persistent calcium oscillations in CCK1R-CHO-K1 cells transfected with human, rat, mouse or Peking duck *CCK1R* gene, which were blocked by ^1^O_2_ quencher Trolox C. After tagging protein photosensitizer miniSOG to C-terminus of these CCK1R, photodynamic action was found to similarly trigger persistent calcium oscillations in CCK1R-miniSOG-CHO-K1 cells expressing human, rat, mouse, and Peking duck receptor constructs. Incubation with Trolox C 300 μM during LED light irradiation also prevented photodynamic CCK1R activation in CCK1R-miniSOG-CHO-K1 cells. In contrast, human M3R was not photodynamically activated with SALPC or tagged miniSOG as the photosensitizer. These data, together, suggest that photodynamic CCK1R activation is maintained outside of the pancreatic acinar cell, making possible photodynamic CCK1R activation in CCK1R-expressing organs and tissues other than the pancreas, with high spatiotemporal precision.

## 1. Introduction

G protein coupled receptors (GPCR) are among the largest families of functional proteins [1,2]. GPCR ligands account for a predominant proportion of all clinically available drugs [3,4]. The large repertoires of small molecule ligands for GPCR are relatively well-explored for pharmacology and therapeutics against human and animal diseases [4,5], but ligand-independent pharmacology of different sub-families of GPCR, however, remains poorly investigated, let alone with any spatial or temporal precision [6].

Ligand-independent GPCR regulation typically includes temperature regulation [7,8,9,10,11], regulation by the lipid microenvironment of the plasma membrane such as cholesterol content [12,13,14], by transmembrane potential [15,16,17,18,19], and regulation by localized concentration of reactive oxygen species such as the lowest lying excited state of molecular oxygen, the delta singlet oxygen (Δ^1^O_2_) [6,20,21,22,23,24,25,26,27,28,29].

Most interestingly, cholecystokinin 1 receptor (CCK1R), a member of the A-class GPCR predominantly expressed at the basolateral plasma membrane in pancreatic acinar cells, has been shown to be activated permanently by ^1^O_2_ generated via type II photodynamic action, with inherent spatial and temporal precision, but with the antagonist-binding site remaining fully intact after photodynamic activation [6,21,23,24,25,26,27,28,29,30,31]. CCK1R both in the freshly isolated mammalian (rat, mouse), avian (Peking duck) pancreatic acini [21,31], and in the rat pancreatic acinar tumor cell line AR4-2J could be readily activated via type II photodynamic action, with either photosensitizer dye (such as sulphonated aluminum phthalocyanine, SALPC) [21,23] or with genetically encoded protein photosensitizers (GEPP) (such as KillerRed, miniSOG) after a brief pulse of light irradiation (1–2 min) [23,25,26,27,28]. The detailed molecular mechanisms for such permanent photodynamic activation of CCK1R are being elucidated (reviewed in [6,22,29,32,33,34]) and the list of GPCR permanently activated by photodynamic action is expanding [35].

The agonist-stimulated activation of CCK1R receptors are known to regulate vital physiological functions [36,37]. The human, rat, mouse, chicken, and Peking duck CCK1R all regulate satiety sensation and feeding, gallbladder contraction, gastric emptying, neurodevelopment, digestive enzyme secretion, inflammation, muscle growth and body weight gain, and other functions [38,39,40,41,42,43,44,45,46,47,48,49,50,51,52,53,54,55,56,57,58,59,60,61]. Although CCK1R is expressed and functions in numerous organs and tissues other than the exocrine pancreas, it is not known whether CCK1R outside of the microenvironment of the pancreatic acinar cell would maintain the special property of being activated by photodynamic action, bearing in mind that the plasma membrane environment, such as cholesterol content, has a major effect on CCK1R activation by agonist stimulation with CCK octapeptide [12,13,14].

The aim of the present work was, therefore, to investigate whether CCK1R expressed outside of the microenvironment of the pancreatic acinar cell would maintain this special property of permanent photodynamic activation. The photodynamic activation of the human, rat, mouse, and Peking duck was therefore examined after ectopically expression in CHO-K1 cells (the alternative is to try to isolate and examine each and every CCK1R-expressing native cell type). Data obtained indicate that photodynamic action with SALPC, or with miniSOG tagged to C-terminus of CCK1R, activated both the free-standing CCK1R and miniSOG-tagged CCK1R (construct CCK1R-miniSOG), confirming that photodynamic CCK1R activation is possible outside of the microenvironment of the pancreatic acinar cell. In parallel experiments, the human M3R was found not photodynamically activated with SALPC or with tagged miniSOG as the photosensitizer.

## 2. Results

### 2.1. Agonist-Stimulated Activation of Ectopically Expressed Human, Rat, Mouse and Peking Duck CCK1R

**Figure 1:**
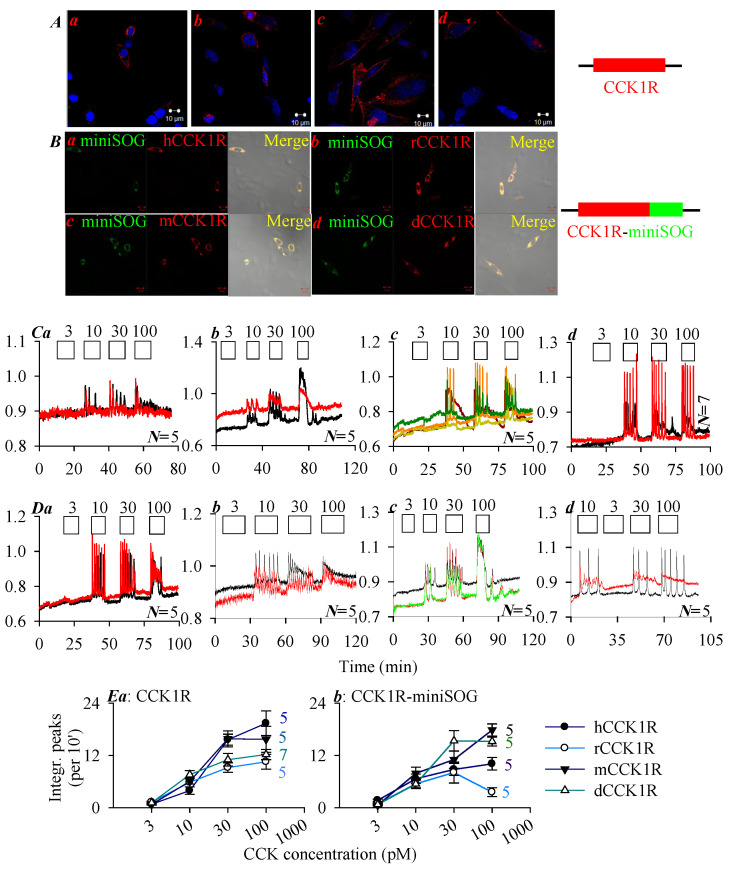
Human, rat, mouse, Peking duck CCK1R and their miniSOG constructs CCK1R-miniSOG were expressed and stimulated with CCK octapeptide. Immunocytochemsitry is shown in panels (**A**,**B**), whereas calcium imaging traces are shown in panels (**C**,**D**). (**A**) CCK1R expression: (**a**) hCCK1R, (**b**) rCCK1R, (**c**) mCCK1R, (**d**) dCCK1R. (**B**) CCK1R-miniSOG expression: (**a**) hCCK1R-miniSOG, (**b**) rCCK1R-miniSOG, (**c**) mCCK1R-miniSOG, (**d**) dCCK1R-miniSOG. Twenty-four (24) hours after transfection, the transfected cells were processed for immunocytochemistry. The fixed and attached cells were incubated sequentially with primary rabbit anti-CCK1R antibody and TRITC-conjugated donkey anti-rabbit secondary antibody; the expression of CCK1R (**A**(**a**–**d**): TRITC, λ_ex_ 543 nm; Hochest 33342, λ_ex_ 405 nm), and CCK1R-miniSOG (**B**(**a**–**d**): TRITC, λ_ex_ 543 nm; miniSOG, λ_ex_ 488 nm) were verified, as seen in the merged images (**B**(**a**–**d**)). Scale bars: 10 μm. CHO-K1 cells were transfected with plasmid *pCCK1R*-3.1^+^ (**A**) or *pCCK1R*-*miniSOG*-3.1^+^ (**B**). Twenty-four (24) h after transfection, CCK1R- or CCK1R-miniSOG-CHO-K1 cells were loaded with Fura-2 AM, attached to the bottom cover-slip of Sykes-Moore perfusion chambers, and perifused. CCK at 3, 10, 30, and 100 pM were added, as indicated by the horizontal bars. (**C**(**a**–**d**)) CHO-K1 cells expressing the human, rat, mouse, and Peking duck CCK1R, respectively. (**D**(**a**–**d**)) CHO-K1 cells expressing construct CCK1R-miniSOG, with CCK1R of the human, rat, mouse, and Peking duck origin. The calcium traces shown in (**C**,**D**) are from one typical experiment out of *N* identical experiments (*N* = 5–7), with each color-coded trace representing one individual cell. (**E**) Dose–response curves of CCK stimulation in CCK1R-CHO-K1 (**a**) and CCK1R-miniSOG-CHO-K1 (**b**) cells, with integrated calcium peak areas above baseline (per 10 min) calculated. (**E**(**a**)) CCK1R. (**E**(**b**)) CCK1R-miniSOG.

CHO-K1 cells transfected with plasmid *pCCK1R-cDNA3.1*^+^ containing the *CCK1R* gene or plasmid *pCCK1R-miniSOG-cDNA3.1*^+^ containing fused construct CCK1R-miniSOG (Figure 1A,B) were processed for immunocytochemistry. Confocal imaging revealed plasma membrane CCK1R localization (red, Figure 1A) or localization of construct CCK1R-miniSOG (yellow) containing CCK1R (red) and miniSOG (green) (Figure 1B).

To examine whether the human, rat, mouse, and Peking duck CCK1R ectopically expressed in CCK1R-CHO-K1 and CCK1R-miniSOG-CHO-K1 cells retained their function, these cells were stimulated with CCK octapeptide (Figure 1C,D). Since CCK1R is known to be coupled to the Gq—phospholipase C—inositol trisphosphate (IP_3_)—IP_3_ receptor (IP_3_R)—Ca^2+^ signaling pathway, CCK1R activation was monitored by imaging cytosolic calcium concentration [2,22,30,32,36]. Stimulation with CCK 3, 10, 30, and 100 pM was found to elicit concentration-dependent calcium increases in CCK1R-CHO-K1 cells expressing the human, rat, mouse, and Peking duck receptor (Figure 1C(a–d)), and also in CCK1R-miniSOG-CHO-K1 cells expressing the human, rat, mouse, and Peking duck receptor construct CCK1R-miniSOG (Figure 1D(a–d)). Very clear CCK dose–response curves were found (calcium peak area above baseline) (Figure 1E). The dose–response curves for both free-standing human, rat, mouse, Peking duck CCK1R (Figure 1E(a)) and for the corresponding CCK1R-miniSOG construct (Figure 1E(b)) are shown. The minimum effective CCK concentrations used to trigger regular calcium oscillations in CCK1R-CHO-K1 and CCK1R-miniSOG-CHO-K1 cells were found to remain in the low picomolar range (Figure 1).

The susceptibility of the ectopically expressed human, rat, mouse, Peking duck CCK1R and their miniSOG-tagged constructs to photodynamic activation, with photosensitizer SALPC and miniSOG respectively, was then examined, as shown below.

### 2.2. Photodynamic Activation of the Human, Rat, Mouse and Peking Duck CCK1R with Photosensitizer SALPC

**Figure 2:**
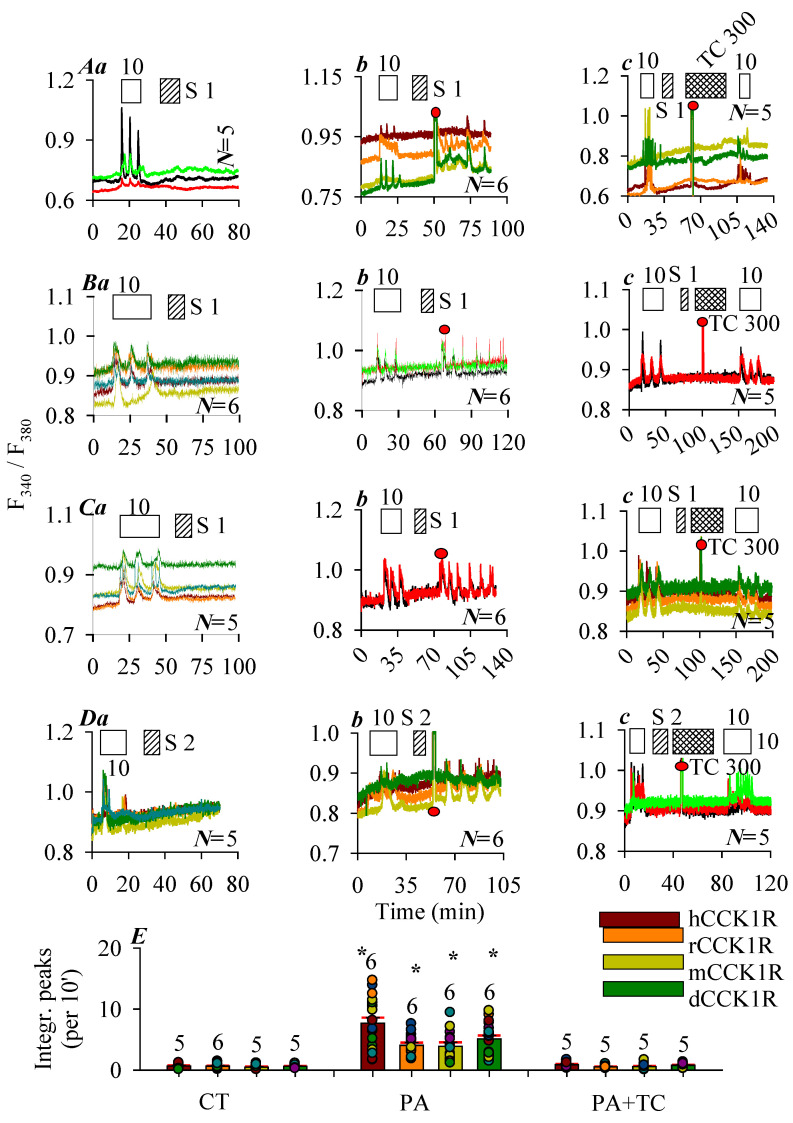
Photodynamic CCK1R activation with SALPC. CHO-K1 cells were transfected with plasmid *pCCK1R* and, 24 h later, CCK1R-CHO-K1 cells were loaded with Fura-2 AM, attached to the bottom cover-slips of Sykes-Moore perfusion chambers, and perifused. CCK (empty bar), SALPC (stippled bar), Trolox C (crossed bar), and red LED (675 nm, 50 mW·cm^−2^, 1.5 min) irradiation were applied as indicated by the horizontal bars: (**A**) Human CCK1R; (**B**), rat CCK1R; (**C**), mouse CCK1R; (**D**), Peking duck CCK1R. In each case, panel (**a**) shows CCK stimulation followed by SALPC; (**b**) CCK stimulation followed by SALPC and LED irradiation; (**c**) CCK stimulation followed by SALPC, LED irradiation in the presence of Trolox C, and finished with a second identical dose of CCK. Note that CCK 10 pM was applied in (**A**–**D**). SALPC 1 μM was applied in (**A**–**C**), 2 μM in (**D**). Trolox C 300 μM was applied in (**A**–**D**). Red LED (675 nm, 1.5 min) (filled red circle: ●) irradiation was at 50 mW·cm^−2^ in (**A**,**C**), 60 mW·cm^−2^ in (**B**), and 40 mW·cm^−2^ in (**D**). The original calcium tracings in panels (**A**–**D**) are each from one typical experiment out of *N* repeats (as indicated in panel), with every color-coded trace representing one individual cell. (**E**) The calcium responses were calculated as integrated peak area per 10 min (integrated peak area during 30 min immediately after red LED irradiation in (**A**–**D**) (**b**,**c**); corresponding time period 30 min in (**A**–**D**) (**a**) were presented. Photodynamic activation (PA) of CCK1R was compared with controls (CT) in the absence of SALPC and LED irradiation, and with photodynamic activation in the presence of Trolox C (PA + TC). Asterisk (*) indicates statistical significance at *p* < 0.05 (*N* = 5–7). Symbols on graph: CT: control; PA: photodynamic action; TC: Trolox C; S: SALPC.

In this series of experiments, photosensitizer SALPC (1 or 2 μM) in the dark was found to have no effect on basal calcium in CCK1R-CHO-K1 cells expressing the human, rat, mouse, and Peking duck CCK1R, although stimulation with CCK 10 pM elicited regular calcium oscillations, which were readily washed out (Figure 2A(a), hCCK1R; Figure 2B(a), rCCK1R; Figure 2C(a), mCCK1R; Figure 2D(a), dCCK1R). After the washing out of SALPC (1, 2 μM), red LED irradiation (675 nm, 50, 60, 50, 40 mW·cm^−2^, 1.5 min) was found to trigger persistent calcium oscillations in CHO-K1 cells positively expressing the human, rat, mouse, and Peking duck CCK1R (Figure 2A(b), hCCK1R; Figure 2B(b), rCCK1R; Figure 2C(b), mCCK1R; Figure 2D(b), dCCK1R). Repeat SALPC (1, 2 μM) photodynamic action (675 nm, 50, 60, 50, 40 mW·cm^−2^, 1.5 min), but in the presence of the ^1^O_2_ quencher Trolox C 300 μM, failed to induce any calcium spikes, although a second dose of CCK 10 pM readily triggered calcium oscillations (Figure 2A(c), hCCK1R; Figure 2B(c), rCCK1R; Figure 2C(c), mCCK1R; Figure 2D(c), dCCK1R). In separate experiments, it was confirmed that Trolox C 300 μM had no effect on calcium oscillations induced by agonist CCK 10 pM in CCK1R-CHO-K1 cells expressing the human, rat, mouse, and Peking duck CCK1R (Figure S1b–e).

Quantification of photodynamically–induced calcium spikes demonstrated clearly that SALPC photodynamic action (SALPC 1 or 2 μM + 675 nm, 60, 50, 45, 40 mW·cm^−2^, 1.5 min) activated the free-standing human, rat, mouse, and Peking duck CCK1R, but these photodynamic activations were blocked by the ^1^O_2_ quencher Trolox C (Figure 2E).

### 2.3. Photodynamic Activation of the Human, Rat, Mouse and Peking Duck CCK1R with Tagged Protein Photosensitizer miniSOG

Effects of photodynamic action with the in-frame miniSOG on human, rat, mouse, and Peking duck CCK1 were examined in CCK1R-miniSOG-CHO-K1 cells expressing construct CCK1R-miniSOG (miniSOG tagged to C-terminus of CCK1R) (Figure 1B). Parental CHO-K1 cells transfected with blank vector *pcDNA3.1^+^* (*pcDNA3.1^+^*-CHO-K1 cells) showed no endogenous CCK1R; CCK of up to 100 nM did not have any effect on basal calcium in these empty vector-transfected *pcDNA3.1^+^*-CHO-K1 cells (Figure S1a).

**Figure 3:**
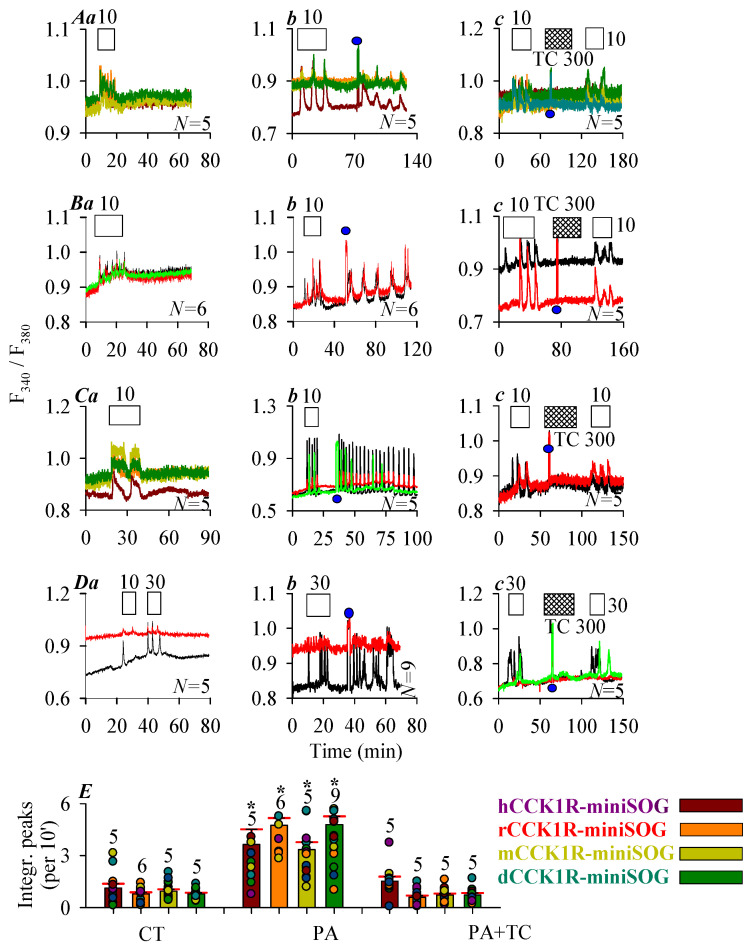
Photodynamic CCK1R activation with C-terminal-tagged miniSOG. CHO-K1 cells were transfected with plasmid *pCCK1R-miniSOG*, with *CCK1R* gene of human, rat, mouse, and Peking duck origin. Twenty-four hours (24 h) after transfection, CCK1R-miniSOG-CHO-K1 cells were loaded with Fura-2 AM, attached to the bottom cover-slip of Sykes-Moore perfusion chambers, and perifused. CCK (empty bar), Trolox C (crossed bar), and blue LED (450 nm, 85 mW·cm^−2^, 1.5 min) (filled blue circle: ●) irradiation were applied, as indicated by the horizontal bars ((**A**), human; (**B**), rat; (**C**), mouse; (**D**), Peking duck). In each case in (**A**–**D**), (**a**) shows CCK stimulation alone; (**b**) CCK followed by a pulse of blue LED light, (**c**) CCK 10 pM followed by a pulse of blue LED light in the presence of Trolox C 300 μM (**A**–**D**), and finally followed by a second identical dose of CCK. Note the different CCK concentrations used: CCK 10 pM in (**A**–**C**), CCK 30 pM (**D**). The color-coded calcium traces shown (each trace from one individual cell) are from one typical experiment out of *N* identical experiments (*N* = 5–9). (**E**) The calcium peak area above baseline (per 10 min) from controls (CT) (from panels (**A**–**D**) (**a**)), photodynamically treated cells (PA) (from panels (**A**–**D**) (**b**)), and photodynamically treated cells in the presence of Trolox C (PA + TC) (from panels (**A**–**D**) (**c**)) were calculated for 30 min immediately after cessation of LED irradiation or the corresponding time period in the absence of light and compared (**A**–**D**). The asterisk (*) indicates statistically significant differences compared with corresponding controls at *p* < 0.05 with Student’s *t*-test.

CCK at 10 or 30 pM were found to elicit robust calcium responses in CHO-K1 cells expressing the human, rat, mouse, and Peking duck receptor construct CCK1R-miniSOG (Figure 3A(a), hCCK1R-miniSOG; Figure 3B(a), rCCK1R-miniSOG; Figure 3C(a), mCCK1R-miniSOG; Figure 3D(a), pdCCK1R-miniSOG). CCK-induced calcium oscillations disappeared immediately after wash out of CCK (Figure 3A(a–c)–D(a–c)), note that subsequent blue LED irradiation (450 nm, 85 mW·cm^−2^, 1.5 min) elicited fresh calcium oscillations which persisted long after cessation of LED light irradiation (Figure 3A(b), hCCK1R; Figure 3B(b), rCCK1R; Figure 3C(b), mCCK1R; Figure 3D(b), dCCK1R). Although ^1^O_2_ quencher Trolox C (300 μM) had no effect on calcium oscillations induced by agonist CCK in CHO-K1 cells expressing the human, rat, mouse, and Peking duck receptor construct CCK1R-miniSOG (Figure S1f–i), in the presence of Trolox C, blue LED irradiation no longer induced any calcium increases, although all subsequent second dose of CCK (10 or 30 pM) triggered regular calcium oscillations (Figure 3A(c), hCCK1R; Figure 3B(c), rCCK1R; Figure 3C(c), mCCK1R; Figure 3D(c), dCCK1R). Quantified calcium response after photodynamic action (450 nm, 85 mW·cm^−2^, 1.5 min) in CCK1R-miniSOG-CHO-K1 cells confirmed activation of human, rat, mouse, and Peking duck CCK1 receptors in construct CCK1R-miniSOG, which was prevented by treatment with Trolox C (Figure 3E).

The above data clearly indicated that photodynamic action with the photosensitizer dye SALPC or with the in-frame protein photosensitizer miniSOG activated human, rat, mouse, and Peking duck CCK1R. Photodynamic CCK1R activation is maintained outside of the plasma membrane microenvironment of the pancreatic acinar cell. For comparisons, we also examined the possible effect of photodynamic action with SALPC or tagged miniSOG on human acetylcholine M3 receptors in parallel experiments.

### 2.4. Lack of Photodynamic Effect on Human M3R

**Figure 4:**
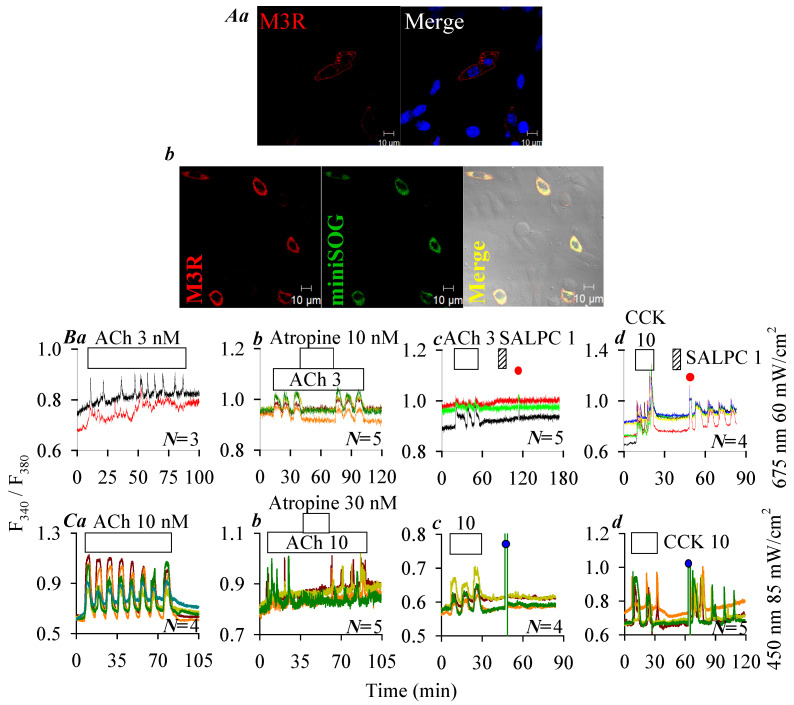
Lack of photodynamic effect on hM3R plasma membrane expression of hM3R (**A**(**a**)), and of fusion protein hM3R-miniSOG (**A**(**b**)). Twenty-four (24) hours after transfection, cells were fixed and attached to cover-slips, incubated sequentially with primary rabbit anti-M3R antibody and TRITC-conjugated donkey anti-rabbit secondary antibody. Expression of M3R (**A**(**a**), λ_ex_: TRITC/543 nm; Hochest 33342, λ_ex_: 405 nm), and of M3R-miniSOG (**A**(**b**), λ_ex_: TRITC/543 nm; λ_ex_: 488 nm) were verified in merged images. Scale bars: 10 μm. hM3R- or rCCK1R-CHO-K1 and hM3R- or CCK1R-miniSOG-CHO-K1 cells loaded with Fura-2 AM were attached to cover-slips forming the bottom part of a Sykes-Moore perfusion chamber, perfused, and exposed to CCK (empty bar), ACh (empty bar), atropine (empty bar) and SALPC (stippled bar) as indicated by the horizontal bars (**B**,**C**): (**B**(**a**)) ACh 3 nM; (**B**(**b**)) ACh 3 nM, atropine 10 nM; (**B**(**c**)) ACh 3 nM, SALPC 1 μM, red light (675 nm, 60 mW·cm^−2^, 1.5 min); (**B**(**d**)) CCK 10 pM, SALPC 1 μM, red light (675 nm, 60 mW·cm^−2^, 1.5 min); (**C**(**a**)) ACh 10 nM, 65 min; (**C**(**b**)) ACh 10 nM, atropine 30 nM; (**C**(**c**)) ACh 10 nM, blue light (450 nm, 85 mW·cm^−2^, 1.5 min); (**C**(**d**)) CCK 10 pM, blue light (450 nm, 85 mW·cm^−2^, 1.5 min). In each panel (**B**(**a**–**d**),**C**(**a**–**d**)), the calcium traces are from one typical experiment out of *N* identical repeats (*N* = 3–5), with each trace representing one individual cell. The time of LED light application is indicated by red (675 nm, ●) or blue (450 nm, ●) filled circles.

Immunocytochemistry performed 24 h after plasmid transfection revealed plasma membrane localization of the human M3R (red) in hM3R-CHO-K1 cells (Figure 4A(a)), and plasma membrane localization of construct hM3R-miniSOG (orange in merged micrograph) containing hM3R (red) and miniSOG (green) in hM3R-miniSOG-CHO-K1 cells (Figure 4A(b)). Note that the intracellular expression of receptor construct M3R-miniSOG seemed to have increased compared to M3R receptor alone (compare Figure 4A(a) and Figure 4A(b)). It would be interesting to investigate such a difference if confirmed true in the future. Since M3R is coupled to the Gq—phospholipase C—inositol trisphosphate (IP_3_)—IP_3_ receptor (IP_3_R)—Ca^2+^ signaling pathway, M3R activation was monitored by imaging cytosolic calcium concentration [6,20,21]. ACh 3 nM triggered regular calcium oscillations in hM3R-CHO-K1 cells (Figure 4B(a)), which were inhibited reversibly with atropine 10 nM (Figure 4B(b)). After wash out of ACh-triggered calcium oscillations in hM3R-CHO-K1 cells, perfusion of SALPC 1 μM or subsequent LED light (675 nm) irradiation (60 mW·cm^−2^, 1.5 min) was found to have no effect on baseline calcium (Figure 4B(c)). However, persistent calcium oscillations appeared under identical photodynamic intensity (SALPC 1 μM, LED 675 nm, 60 mW·cm^−2^, 1.5 min) in rCCK1R-CHO-K1 cells expressing the rat CCK1R in time-matched parallel experiments (Figure 4B(d)). Although both CCK stimulation and SALPC photodynamic action induced calcium oscillations (Figure 4B(d)), the CCK (10 pM) effect was readily washed out, but photodynamically induced calcium oscillations persisted after completion of the 1.5 min duration of light irradiation (Figure 4B(d)).

ACh 10 nM triggered calcium oscillations also in hM3R-miniSOG-CHO-K1 cells expressing the construct hM3R-miniSOG (Figure 4C(a)). ACh-triggered calcium oscillations were blocked reversibly by atropine 30 nM (Figure 4C(b)). Calcium increases elicited by ACh 10 nM were readily washed out in hM3R-miniSOG-CHO-K1 cells, but subsequent LED (450 nm) light irradiation (85 mW·cm^−2^, 1.5 min) had no effect (Figure 4C(c)). In time-matched (same day experiments) parallel experiments, however, it was re-confirmed that after wash out of CCK 10 pM-elicited calcium oscillations, LED light (450 nm) irradiation (85 mW·cm^−2^, 1.5 min) induced persistent calcium oscillations in hCCK1R-miniSOG-CHO-K1 cells expressing the construct hCCK1R-miniSOG (Figure 4C(d)).

Therefore human, rat, mouse, and Peking duck CCK1R could all be photodynamically activated in type II photodynamic action, with either SALPC as the photosensitizer or with miniSOG attached to the C-terminal of the receptor as the photosensitizer. Under identical conditions, the human M3R was not at all affected by photodynamic action.

The protein sequences and structures of the human, rat, mouse, and Peking duck CCK1R and human M3R were then examined to shed possible light on the molecular mechanisms for photodynamic activation of CCK1R or the lack of effect on the M3R.

### 2.5. Structural Correlates of Permanent Photodynamic Activation of the Human, Rat, Mouse and Peking Duck CCK1R

**Figure 5:**
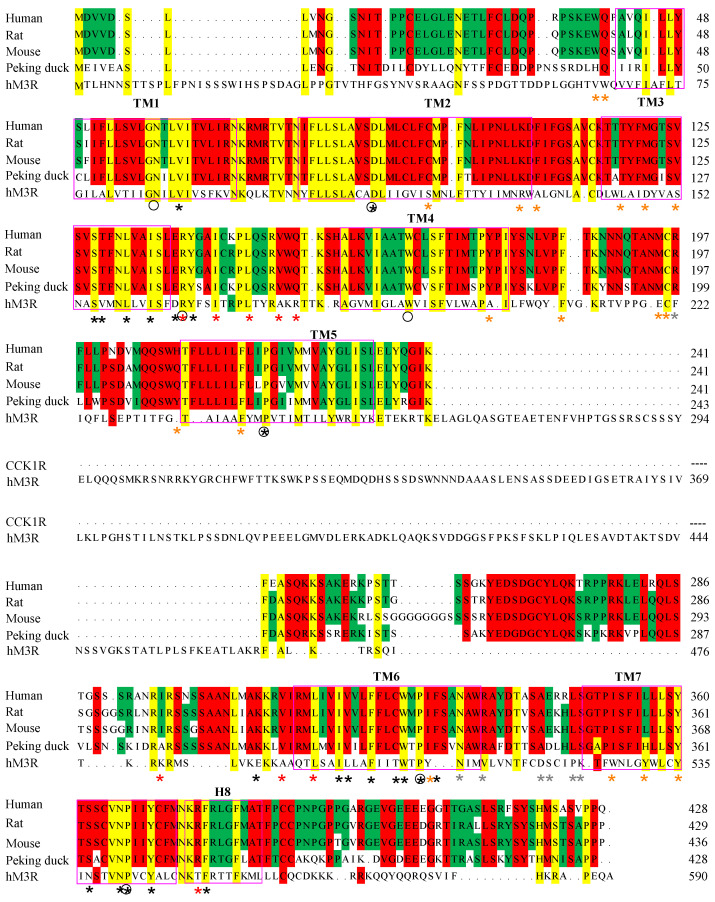
Sequence alignment of CCK1R. The human (*Homo sapiens*), rat (*Rattus norvegicus*), mouse (*Mus musculus*), and Peking duck (*Anas platyrhynchos domestica*) CCK1R full sequences are aligned. hCCK1R (NP_000721.1), rCCK1R (NP_036820.1), mCCK1R (NP_033957.1), and dCCK1R (MN250295.1) were aligned with Mega 7. Asterisks (*) indicate key residues for the activation of hCCK1R by CCK-8S (grey, CCK-8 recognition; brown, CCK-8 binding; black, conformational change; red, G protein binding). Black circles denote Ballesteros–Weinstein reference residues N^1.50^, D^2.50^, R^3.50^, W^4.50^, P^5.50^, P^6.50^, and P^7.50^. Pink rectangles outline TM1-7 and H8, with lettering placed above the sequence. Colored background indicates complete identity of residues.

Examination of the receptor protein sequence revealed that the human, rat, mouse and Peking duck CCK1R all shared the same “Y^3.30^F^3.31^M^3.32^” motif in TM3, which has been found previously to be important for photodynamic activation [27,28]. This motif is lacking in the human M3R; the corresponding residue triplet is A^3.30^I^3.31^D^3.32^ (Figure 5). It is noted that all the higher vertebrate CCK1R also shared an extended version of “YFM” in TM7—“Y^7.53^C^7.54^F^7.55^M^7.56^” (Figure 5, denoted with the Ballesteros-Weinstein numbering system, see legend), whereas in the un-susceptible human M3R, the corresponding sequence is “Y^7.53^A^7.54^L^7.55^C^7.56^” (Figure 5). Of the critical residues identified in early point mutation experiments of human CCK1R (marked with an asterisk * in Figure 5), only C^2.57^, M^3.32^, Y^3.51^, Y^4.60^, M^195.ECL2^, C^196.ECL2^, C^6.47^, W^6.48^, Y^7.43^, and Y^7.53^ are completely conserved in the vertebrate CCK1R examined in this work and also susceptible to ^1^O_2_ oxidation (Figure 5). Note that Y^7.53^ is also part of the “Y^7.53^C^7.54^F^7.55^M^7.56^” motif mentioned above. A cluster of ^1^O_2_-susceptible residues “Y^418^S/T^419^H^420^M^421^” in the C-terminus of the human CCK1R is also found in the rat, mouse, and Peking duck CCK1R; a similar cluster of residues is not found in the human M3R (Figure 5).

**Figure 6:**
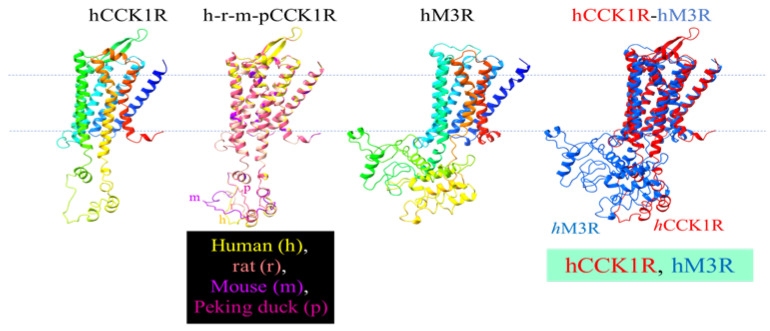
Modeled structure of human, rat, mouse, Peking duck CCK1R, of human M3R, of merged mammalian and avian CCK1R, and of merged human CCK1R and M3R. The 3D structures were generated by Swiss-Model using human CCK1R (PDB: 7F8X) as template. Human CCK1R and M3R are each color gradient-coded (blue-to-red corresponds to N- to C-terminus). Human, rat, mouse, and Peking duck CCK1R are merged (h-r-m-p) with ChimeraX 1.3: human, yellow; rat, brown; mouse, purple; Peking duck, erythritol (a version of merged h-r-m-pCCK1R has been published previously in [31]). The human M3R structure is generated by Swiss-Model using rat M3R (PDB: 4DAJ) as template. Human CCK1R and M3R are merged with ChimeraX 1.3: hCCK1R, red; hM3R, blue.

The solved human CCK1R structure (7F8X) was used as a template to predict the structure of the rat, mouse, and Peking duck CCK1R (Figure 6). The similarities of these human receptor homologs are readily noted. Other than the 7 TM helix, note the short ICL1, ECL1, the twisted ICL2, bipartite beta sheet ECL2, extended ICL3, near horizontal helical ECL3, and helix 8 (Figure 6). Note the overlapping secondary structures in ICL3, with noted variations in the secondary-structure-free region in all these vertebrate structures (Figure 6). A comparison of hCCK1R and hM3R structures reveals that not much deviates among TM1-4, but TM5 in hM3R dips deeply into the cytosol and ICL3 in hM3R is much more extensive (Figure 6). Merged structures of human *h*CCK1R/*h*M3R revealed extensive diversion in ICL3 in the refractory *h*M3R, among other more subtle differences (Figure 6). The beta sheet structure in ECL2 and the horizontal helix in ECL3 in CCK1R are not seen in ECL2 and ECL3 in hM3R, but a more extensive helix 8 C-terminal tail is noted in the human ACh M3R (Figure 6)**.**

## 3. Discussion

In the present work, it was found that the human, rat, mouse, and Peking duck CCK1R ectopically expressed in positive CCK1R-CHO-K1 cells were all photodynamically activated with either perifused photosensitizer SALPC or in CCK1R-miniSOG-CHO-K1 cells with C-terminal-tagged protein photosensitizer miniSOG. The photodynamic CCK1R activation was blocked with ^1^O_2_ quencher Trolox C. Although the CCK1R were all susceptible (with some variations in sensitivity and, therefore, varied LED 450 nm power density was used) to photodynamic activation, the human, rat, mouse, and Peking duck CCK1R showed similar responses towards picomolar CCK stimulation. These data suggest that photodynamic CCK1R activation is maintained outside of the plasma membrane microenvironment of the pancreatic acinar cell.

CCK stimulation of the human, rat, mouse, and Peking duck CCK1R, ectopically expressed as free-standing CCK1R or as construct CCK1R-miniSOG in CHO-K1 cells, triggered calcium oscillation dose-dependently (Figure 1). Tagging with miniSOG did not seem to drastically change CCK1R or CCK1R-miniSOG sensitivity towards CCK stimulation (Figure 1).

It is known that SALPC photodynamic action activated CCK1R permanently both in the isolated rat, mouse, and Peking duck pancreatic acini [21,30,31] and activated human CCK1R ectopically expressed in CCK1R-HEK-293 cells [23]. SALPC (i.e., AlPcS_4_) with central conjugated Al^3+^ and four peripheral sulfonate groups showed good aqueous solubility and high ^1^O_2_ quantum yield [7,62,63,64,65]; both the central Al^3+^ and the peripheral sulfate groups have been found to be needed for photodynamic CCK receptor activation [35]. SALPC had no effect in the dark on baseline calcium in CCK1R-CHO cells expressing the human, rat, mouse, and Peking duck CCK1R (Figure 2A(a)–D(a)); but subsequent red LED light irradiation (675 nm, 60, 50, 40 mW·cm^−2^) triggered persistent calcium oscillations (Figure 2A(b)–D(b)); in the presence of the ^1^O_2_ quencher Trolox C, red LED light irradiation (675 nm, 60, 50, 40 mW·cm^−2^) no longer had any effect (Figure 2A(c)–D(c)). These data are similar to previous works with the human CCK1R [23,25,26,27,28]. This would suggest that all vertebrate CCK1R, ectopically expressed outside of the pancreatic acinar cell plasma membrane microenvironment, could be photodynamically activated with SALPC as the photosensitizer, with ^1^O_2_ being the predominant reactive intermediate.

The genetically encoded protein photosensitizer miniSOG (λ_ex_ 448 nm, λ_em_ 500 nm), with a ^1^O_2_ quantum yield of ≥0.03 ± 0.01 [66], could effectively activate the human CCK1R photodynamically [23,25,26]. The present work found that miniSOG fusion to the C-terminal of CCK1R did not seem to change the receptor sensitivity drastically towards CCK stimulation, for all vertebrate CCK1R examined in this work (Figure 1). CCK stimulation induced calcium oscillations in all vertebrate receptor constructs in CCK1R-miniSOG-CHO-K1 cells, which were readily washed out (Figure 3A(a)–D(a)); LED (450 nm, 85 mW·cm^−2^) irradiation then induced fresh calcium oscillations (Figure 3A(b)–D(b)); In the presence of ^1^O_2_ quencher Trolox C, LED light irradiation (450 nm) no longer induced any calcium oscillations with all vertebrate CCK1R-miniSOG in transfected CHO-K1 cells (Figure 3A(c)–D(c)); The vitamin E analog of Trolox C, as ^1^O_2_ quencher in the μM to mM range, has been used before to block the effect of ^1^O_2_ [67,68,69]; we could confirm that Trolox C had no effect on CCK stimulated receptor activation and calcium oscillations (Figure S1).

Parallel experiments found that although the human hM3R was not affected by SALPC photodynamic action, with either SALPC or C-terminal-tagged miniSOG as the photosensitizer, the rat rCCK1R was readily activated in SALPC photodynamic activation (Figure 4). The human receptor construct hM3R-miniSOG was not photodynamically activated when the human receptor construct hCCK1R-miniSOG was readily photodynamically activated (Figure 4). These data confirm that neither the free-standing hM3R nor the hM3R-miniSOG construct share the property of photodynamical activation which is unique to human, rat, mouse, or Peking duck CCK1R, either as stand-alone CCK1R or as CCK1R-miniSOG construct (Figure 2 and Figure 3). It may be noted here that both vertebrate CCK1R (Figure 2, Figure 3 and Figure 4 in this work) and human CCK2R [35] are photodynamically activated with tagged protein photosensitizer miniSOG, although it is known that only agonist-stimulated activation of the human CCK1R is affected by plasma membrane cholesterol content whereas agonist-stimulated activation of the human CCK2R is not affected by cholesterol content [12,13,14,70]. This suggests that cholesterol oxidation might not play a role in the photodynamic activation of CCK1R and CCK2R, although singlet oxygen oxidation fingerprints have been established for plasma membrane cholesterol [71]. This is indirectly supported by the fact that although Y^3.51^ is critical for cholesterol binding and cholesterol sensitivity of agonist-stimulated activation of the human CCK1R [14], this Y^3.51^ is part of the ERY motif in CCK1R of all high vertebrates and of the DRY motif in the refractory human ACh M3R (Figure 5). In this context, it may be noted that while agonist-stimulated activation of CCK1R is affected by cholesterol and sphigolipid content, some other A-class GPCR are not [12,14,70,72]. It would be interesting, in the future, to investigate whether other plasma membrane physicochemical properties such as membrane viscosity, which is known to facilitate secretory protein accumulation at the endoplasmic reticulum (ER) exit sites (ERES) but attenuate transport to the Golgi apparatus [73], would affect photodynamic CCK1R activation at the plasma membrane. Other than plasma membrane-delimited expression, we also detected some intracellular expression of both CCK1R (Figure 1A) and M3R (Figure 4A(a)) and their constructs (CCK1R-miniSOG, Figure 1B, and M3R-miniSOG, Figure 4A(b)). This could mean that both receptors were in transit to the plasma membrane after synthesis at the endoplasmic reticulum or recycled to endosomes after activation at the plasma membrane. Whether intracellular CCK1R would play a role distinct from the cell surface receptor will be an interesting topic to further investigate in the future. For this matter, photodynamic activation of intracellular CCK1R with a tagged protein photosensitizer would offer obvious advantages over ligand-dependent receptor pharmacology, because no agonists would then be needed to cross the plasma membrane barrier and diffuse to the targeted intracellular receptor, as shown previously [26].

The human, rat, mouse, and Peking duck CCK1R protein sequence and structure are compared with the human M3R (Figure 5 and Figure 6) to shed light on the molecular mechanisms for photodynamic activation of CCK1R or lack of effect on M3R.

For agonist-stimulated activation of the human CCK1R, CCK-binding residues include those in the extracellular loops 1-3 (ECL1-3) (F^107.ECL1^, M^195.ECL2^, R^197.ECL2^, L^347.ECL3^, S^348.ECL3^), in hydrophobic cavities beneath the ECL (F^107.ECL1^, C^196.ECL2^, T^118/3.29^, M^121/3.32^), and in lower (towards intracellular space) parts of the TM domains (I^210/5.39^, N^333/6.55^, R^342/6.58^, Y^7.43^) [74,75,76]. CCK stimulation of CCK1R showed slight variations in sensitivity in the human, rat, mouse, and Peking duck receptors (Figure 1). Note that Cys^2.57^ and Trp^6.48^ are conserved in all these high vertebrate species, to stabilize the CCK1R structure (Figure 5). The ligand-binding pocket in the M3R crystal structure is composed of D^3.32^, S^3.36^, N^3.37^, T^5.42^, L^225.ECL2^, N^6.52^, K^523.ECL3^, and Y^7.43^; the bound ligand tended to be deeply embedded in the TM core spiral bundles and covered by conserved residues Y^3.33^, Y^6.51^, Y^7.39^ [77,78,79,80,81]. The M3R ligand-binding pocket contains only one residue Y^7.43^ which could be oxidized by ^1^O_2_. For CCK1R, the agonist CCK-binding pocket contains not only Y^7.43^, but also other ^1^O_2_-susceptible residues (C^2.57^, M^3.32^, M^195.ECL2^, W^6.48^) (Figure 5).

For photodynamic CCK1R activation, we need to examine only critical residues that are also susceptible to ^1^O_2_ oxidation. Among the ^1^O_2_-susceptible residues Met, Trp, Cys, His, and Tyr [22,82,83], Met oxidation and Met(O) reduction by methionine sulfoxide reductase (Msr), in particular, has been associated with finely tuned functional protein activities and related physiological functions [33]. Permanent photodynamic CCK1R activation by ^1^O_2_ with both SALPC and miniSOG may be associated with residues critical for agonist-stimulated CCK1R activation [84,85,86,87,88]. Such critical residues also susceptible to ^1^O_2_ oxidation are Cys^94/2.57^, Met^121/3.32^, Met^195.ECL2^, Trp^326/6.48^, and Tyr^360/7.43^. Of these five residues, Cys^94/2.57^, Trp^326/6.48^, and Tyr^360/7.43^ are conserved in the human, rat, mouse, and Peking duck CCK1R (Figure 5).

We have found previously that TM3, especially the Y^3.30^F^3.31^M^3.32^ motif in the human CCK1R, is a pharmacophore for photodynamic CCK1R activation in fusion construct miniSOG-CCK1R [28]. Alignment of TM3 sequences of CCK1R revealed a consensus sequence of Y^3.30^F^3.31^M^3.32^ in the human, rat, mouse, and Peking duck CCK1R; the corresponding motif in the refractory human ACh M3R is A^3.30^I^3.31^D^3.32^ (Figure 5).

Vertebrate CCK1R structures modeled after the human structure show almost complete overlap, but note the varied size of ICL3 of CCK1R from different species, as noted previously (Figure 6) [31]. Five structural motifs critically involved in agonist-stimulated activation of GPCR, including the human CCK1R, are as follows: Na^+^ pocket [D^87/2.50^S^128/3.39^S^332/7.45^N^366/7.49^], transmission switches [C^325/6.47^W^326/6.48^xP^328/6.50^] and [P^221/5.50^T^129/3.40^F^322/6.44^], ionic lock switch [E^138/3.49^R^139/3.50^Y^140/3.51^], ionic lock [R^139/3.50^ to K^6.30^], and Tyr toggle switch [N^366/7.49^P^367/7.50^xxY^370/7.53^] [76,89,90,91]. The DSSN, CWxP, ERY, RK, and NPxxY motifs for agonist-stimulated human CCK1R activation are identical to the CCK1R of rat, mouse, and Peking duck. Although it is not known yet whether these motifs involved in agonist-stimulated GPCR activation are important for photodynamic CCK1R activation, the ^1^O_2_-susceptible Met^121/3.32^ and Met^195.ECL2^, as mentioned above, might be relevant due to the sulfur–aromatic interactions of Met.

Met interacts with aromatic residues within a distance of 7 Å, known as sulfur–aromatic interaction [92]. Met residue may bridge with one or two aromatic residues [93], such as F31-M72-Y19 in human estrogen receptor [94], W578-M585-Y436 in lipoxygenase (at 3.8 Å, 3.9 Å) [95], M144-F141 in calmodulin [96], F214-M181-Y198 in inositol polyphosphate kinase (PDB: 6C8A) (F214-M181 at 4.6 Å; Y198-M181 at 4.5 Å), and Y34-M37-F55 in human ferritin (PDB: 2FHA) [94], to stabilize the protein structures or to facilitate ligand–receptor binding [92,97].

In the human CCK1R-antagonist complex structure, aromatic residues within 7 Å from M^3.32^ include Y^7.43^, W^6.48^, and F^6.52^; therefore, interactions of W^6.48^-M^3.32^-W^6.52^ [91] may exist in the resting CCK1R (Figure 5). In CCK-activated human CCK1R [76], the distance between M^3.32^ and the nearby aromatic residues increased, interaction between M^3.32^ and W^6.48^ or F^6.52^ disappeared, and M^3.32^ interaction with Y^7.43^ also weakened, possibly due to the binding of G protein [91]. Oxidized Met becomes more hydrophilic, increasing the Met-aromatic bridge strength by 0.5–1.4 kCal/mol [96]. Met^144^ replacement by glutamine (Q) in calmodulin (to simulate oxidation) is known to increase the frequency of native interaction between M/Q^144^ and F^141^ from 12% to 49%, decreasing the distance from 11.2 to 5.1 Å [96], whereas Met^109^ replacement in calmodulin by glutamine (Q) increases the interactions of M^109^-F^141^ and M^124^-F^141^ to stabilize the Ca^2+^-saturated state of calmodulin [98]. Met^230^ oxidation of cytochrome C peroxidase is known to result in a stronger interaction of Met^230^-Trp^191^ (distance between Met and W pyrrole ring C atom decreasing by 0.2 Å) [93]. M^3.32^ oxidation of CCK1R may similarly result in enhanced interactions between M^3.32^ and W^6.48^ or F^6.52^. Such covalent changes would be different from agonist-stimulated receptor activation, therefore leading to permanent photodynamic activation.

The CCK1R protein isolated from rat pancreatic acini was found previously to be converted from dimer to monomer, near quantitatively, after SALPC photodynamic oxidative activation [24]. TM6 is known to be important for the formation of CCK1R dimers due to the existence of a hydrophobic interface consisting of aliphatic I^6.38^, V^6.42^, and L^6.46^ [99]. The corresponding M^6.38^, I^6.42^, and L^6.46^ in Peking duck CCK1R may imply that the Peking duck CCK1R be slightly different in terms of receptor dimerization from mammalian CCK1R (Figure 5). Met oxidation to MetO by ^1^O_2_ generated in type II photodynamic action may strengthen interactions between Met^3.32^ and W^6.48^ or F^6.52^, this two-bridge interaction (W^6.48^-Met^3.32^-F^6.52^) might break the nearby hydrophobic interface formed by the hydrophobic residues (I^6.38^, V^6.42^, L^6.46^) between two CCK1R protein molecules, to promote monomer formation, at least in the human, rat, and mouse CCK1R. Importantly, other than Met^3.32^, W^6.48^ and F^6.52^ are also conserved in the human, rat, mouse, and Peking duck CCK1R, and the three aliphatic residues at the corresponding positions of CCK1R are human, rat, and mouse—IVL, Peking duck—MIL. Other than the aromatic F, all other residues are hydrophobic (aliphatic) residues. Met^3.32^ photodynamic oxidation or evolution to Q^3.32^ would help to break such hydrophobic interface, resulting in CCK1R dimer-to-monomer conversion that has been observed experimentally with rat pancreatic acinar cell CCK1R [24]. Further, the varied length of disordered region in ICL3 of the human, rat, mouse, and Peking duck CCK1R may exert a graded effect on the monomerization process, related to the disordered region-regulated liquid phase separation of proteins [31,100]. In sharp contrast to the agonist stimulation- and photodynamic action-induced CCK1R monomerization [24], muscarinic receptor agonist carbachol seems to stimulate dimerization/oligomerization of both human [101] and rat [102] muscarinic ACh M3R.

In conclusion, the human, rat, mouse, and Peking duck CCK1R are all photodynamically activated outside of the pancreatic acinar cell plasma membrane microenvironment. In contrast, the human ACh M3R are unaffected by photodynamic action. It is noteworthy that, other than M(Q)^121/3.32^, M(K/T)^195.ECL2)^, ^1^O_2_-susceptible critical residues C^2.57^, W^6.48^ and Y^7.43^ are all conserved in all these vertebrate species examined. The Y^3.30^F^3.31^M^3.32^ triplet in the human, rat, mouse, and Peking duck CCK1R is of particular interest. The fact that permanent photodynamic activation of CCK1R is maintained outside of the plasma membrane microenvironment of the pancreatic acinar cells suggests that photodynamic activation of CCK1R could be used to study CCK1R physiology, pharmacology, and probably therapeutics in organs and tissues other than the exocrine pancreas, with inherent spatiotemporal precision and specificity that are associated with genetically encoded protein photosensitizers, genetically guided photobiophysics [103,104,105], and other related biotechnologies [106,107,108,109].

## 4. Materials and Methods

### 4.1. Materials

Sulfated cholecystokinin octapeptide (CCK, #1166) was from Tocris Cookson (Bristol, UK). MEM amino acid mixture (50×, #11130051), trypsin 0.25% (#25200056), fetal bovine serum (FBS, #10099141), and cell culture medium mix DMEM/F12 (#11320033) were from Thermo Scientific (Shanghai, China). Fura-2 AM (#21020) was from AAT Bioquest (Sunnyvale, CA, USA). Goat anti-CCK1R antibody (#ab77269) and tetramethylrhodamine isothiocyanate (TRITC)-conjugated donkey anti-goat secondary antibody (#ab6738), rabbit anti-M3R primary antibody (#ab41169), and TRITC-conjugated donkey anti-rabbit secondary antibody (#ab7080) were all from Abcam (Cambridge, UK). JetPRIME^®^ transfection reagent (#114-07) was from Polyplus-Transfection (Illkirch, France). Cell-Tak (#354241) was from BD Bioscience (Bedford, MA, USA). Plasmid extraction kit (#4992422) was from TianGen Biochemicals (Beijing, China). Sulfonated aluminum phthalocyanine (SALPC, #AlPcS 834) was from Frontier Scientific (West Logan, UT, USA). Hoechst 33342 (#H342) was from DojinDo (Beijing, China). Plasmids *pcDNA3.1*^+^/*hCCK1R* (GenBank accession number AY322549, #CCKAR00000) and *pcDNA3.1*^+^/*hM3R* (GenBank accession number X15266, (#MAR00000) were from cDNA Resource Center (Rolla, MO, USA). HiPure Total RNA Plus Mini Kit (#R4121-02) was from Magen (Guangzhou, China). 2×Taq Master Mix (#P112-01) was from Vazyme (Nanjing, China). GoScript Reverse Transcription Kit (**#**238813) was from Promega (Shanghai, China). Trolox C (#238813), acetylcholine (ACh, #A6625) and soybean trypsin inhibitor (#T9128) were from Sigma (St. Louis, MO, USA). GoldView (#GV-2) was from SaiBaiSheng (Beijing, China).

Ringer’s buffer had the following composition (in mM): NaCl 118, KCl 4.7, CaCl_2_ 2.5, MgCl_2_ 1.13, NaH_2_PO_4_ 1.0, D-glucose 5.5, HEPES 10, L-glutamine 2.0, and BSA 20 g·L^−1^, MEM amino acid mixture (50×) 2%, soybean trypsin inhibitor 0.1 g·L^−1^, pH adjusted to 7.4 with 4 M NaOH and oxygenated with pure O_2_. BSA, amino acid mixture, and soybean trypsin inhibitor were omitted for perifusion during calcium imaging.

### 4.2. Cell Culture

Chinese hamster ovary K1 (CHO-K1) cells purchased from Shanghai Institute of Life Sciences, Chinese Academy of Sciences were cultured in DMEM/F12 (1:1), supplemented with 10% fetal bovine serum (FBS, Gibco, Shanghai, China) in a CO_2_ incubator under 5% CO_2_ at 37 °C.

### 4.3. Vector Constructs and CHO-K1 Cell Transfection

The human CCK1R and human M3R plasmids *phM3R pcDNA3.1*^+^/*hCCK1R* and *pcDNA3.1*^+^/*hM3R* were bought from the cDNA Resource Center (Bloomsburg, PA, USA). The rat *rCCK1R* (NM_012688.3), mouse *mCCK1R* (NM_009827.2), and Peking duck *dCCK1R* (MN250295.1) genes were mammalian code-optimized and synthesized by Genscript (Nanjing, China) for the construction of plasmid with *pCCK1R-3.1^+^*.

Mammalian codon-optimized *miniSOG* (GenBank accession number JX999997) was synthesized from nucleotides (Genscript, Nanjing, China). The *miniSOG* sequence: ATGGAGAAGTCTTTCGTGATCACCGACCCCAGGCTGCTGATAACCCAATCATCTT CGCCTCCGACGGCTTTCTGGAGCTGACAGAGTACAGCCGGGAGGAGATCCTGGG CAGGAATGGCCGGTTTCTGCAGGGCCCCGAGACCGATCAGGCCACAGTGCAGA AGATCAGAGACGCTATCAGAGATCAGCGCGAGATCACCGTGCAGCTGATCAAC TACACAAAGTCCGGCAAGAAGTTCTGGAATCTGCTGCACCTGCAGCCCATGCGC GACCAGAAGGGCGAGCTGCAGTACTTCATCGGCGTGCAGCTGGATGGCTGA. For the construction of fused plasmids *pCCK1R-miniSOG*, *phM3R-miniSOG*, this *miniSOG* sequence was amplified and ligated to 3′ end of the receptor (*CCK1R*, *M3R*) open reading frame in vectors *pCCK1R*-*3.1*^+^ or *phM3R* (Genscript, Nanjing, China).

Plasmids obtained as described above were used to transfect CHO-K1 cells. A predetermined amount of each plasmid (2 µg DNA) was mixed in transfection buffer (200 µL) before addition of the transfection reagent (jetPRIME^®^, 4 µL of stock). The mixture was allowed to sit for 10 min at room temperature before use. CHO-K1 cells were planted in each culture plate (35 mm) and, 24 h later, 200 µL of the above transfection mixture (containing plasmid and transfection reagent jetPRIME^®^) was added. Transfected CHO-K1 cells were used for immunocytochemistry and for calcium imaging 24 h after transfection.

### 4.4. Immunocytochemistry

Parental CHO-K1 cells were planted on the glass cover-slips and cultured overnight before transfection, as shown above. At 24 h after transfection, CHO-K1 cells expressing the human, rat, mouse, Peking duck CCK1R or human, rat, mouse, Peking duck receptor construct CCK1R-miniSOG, human M3R, human receptor construct M3R-miniSOG were fixed, permeabilized, and blocked for immunocytochemistry. Cells were counter-stained with Hoechst 33342 for 15 min after incubation with secondary antibodies. Stained cells were imaged in a confocal microscope (Zeiss LSM710, Jena, Germany), under oil objective 63×/1.40, with λ_ex_ for Hoechst 33342 at 405 nm; λ_ex_ for TRITC (CCK1R or M3R) at 543 nm; and λ_ex_ for miniSOG at 488 nm.

### 4.5. Photodynamic Treatment

Parental CHO-K1 cells, CCK1R-CHO-K1, M3R-CHO-K1 cells were incubated with photosensitizer SALPC (1 or 2 μM, 10 min), before washing out and irradiation with red LED 675 nm (60, 50, 45, 40 mW·cm^−2^, 1.5 min). Parental CHO-K1 cells, CCK1R-miniSOG-CHO-K1, and hM3R-miniSOG-CHO-K1 cells were irradiated with blue LED 450 nm (85 mW·cm^−2^, 1.5 min).

LED light source was from LAMPLIC, with appropriate light head (675 nm or 450 nm) attached (Shenzhen, China). The irradiation light power density used was determined at the level of attached cells in the Sykes-Moore perfusion chamber with a power meter as reported previously (IL1700, International Light Inc., Newburyport, MA, USA) [25,26,27,28,31].

### 4.6. Calcium Imaging

Cells were loaded with Fura-2 AM (10 µM) in a shaking bath (37 °C, 30 min, 50 cycles per min) and then attached to the bottom cover-slip (coated with Cell-Tak, 1.74 g·L^−1^, 3 µL on each cover-slip) of Sykes-Moore perfusion chambers for at least 30 min before perifusion and experimentation.

The cell-attached perifusion chamber was placed on the platform of a Nikon NE 3000 inverted fluorescence microscope connected to the calcium imaging device (Photon Technology International, PTI, Edison, NJ, USA) with alternating excitations at 340 nm/380 nm (monochromator DeltaRam X, PTI). Emission (emitter 510 ± 40 nm) was detected with a CCD (NEO-5.5-CL-3, Andor/Oxford Instruments, Belfast, UK). The fluorescence ratios F_340/_F_380_ indicative of cytosolic calcium concentrations were plotted against time with SigmaPlot (version 15.0), as reported previously [21,24,25,26,27,28,31,110,111,112,113].

### 4.7. Data Analyses

All data are presented as mean ± SEM (standard error of means). The Student’s *t*-test was performed for comparison between control and experimental groups. Statistical significance at *p* < 0.05 is indicated by an asterisk (*).

## Data Availability

The original contributions presented in this study are included in the article/Supplementary Materials. Further inquiries can be directed to the corresponding author.

## Supplementary Materials

The following supporting information can be downloaded at: https://www.mdpi.com/article/10.3390/ijms262412011/s1.

## Abbreviations

ACh	Acetylcholine
AlPcS4	Sulphonated (4) aluminum phthalocyanine
CCD	Charge coupled device
CCK1R	Cholecystokinin 1 receptor
CHO	Chinese hamster ovary cells
dCCK1R	Duck cholecystokinin 1 receptor
ECL	Extracellular loop
ER	Endoplasmic reticulum
ERES	Endoplasmic reticulum exit sites
FBS	Fetal bovine serum
GPCR	G protein coupled receptor
GPCR-ABSO	G protein coupled receptor activated by singlet oxygen
GEPP	Genetically encoded protein photosensitizer
hCCK1R	Human cholecystokinin 1 receptor
HEK293	Human embryonic kidney cell line 293
hM3R	Human type 3 muscarinic acetylcholine receptor
ICL	Intracellular loop
LED	Light emission diode
M3R	Type 3 muscarinic acetylcholine receptor
miniSOG	mini singlet oxygen generator
mCCK1R	Mouse cholecystokinin 1 receptor
Msr	Methionine sulfoxide reductase
MEM	Minimum essential medium
PDB	Photodynamic biology; Protein Data Bank
PTI	Photon Technology International Inc.
^1^O_2_	Singlet oxygen
pCCK1R	Peking duck cholecystokinin 1 receptor
rCCK1R	Rat cholecystokinin 1 receptor
ROS	Reactive oxygen species
SALPC	Sulphonated aluminum phthalocyanine
SEM	Standard error of means
TM	Transmembrane domain
TRITC	Tetramethylrhodamine isothiocyanate
