# Supplementary material for: Photodynamic Activation of Mammalian and Avian Cholecystokinin Type 1 Receptor Outside of the Pancreatic Acinar Cell Microenvironment
Source: Int J Mol Sci. 2025 Dec 13;26(24):12011. doi: 10.3390/ijms262412011 (PMC12733227; doi:10.3390/ijms262412011)
Supplement: Supplementary file 1 [file ijms-26-12011-s001.zip › ijms-3976085-supplementary.pdf]

Supplemental information

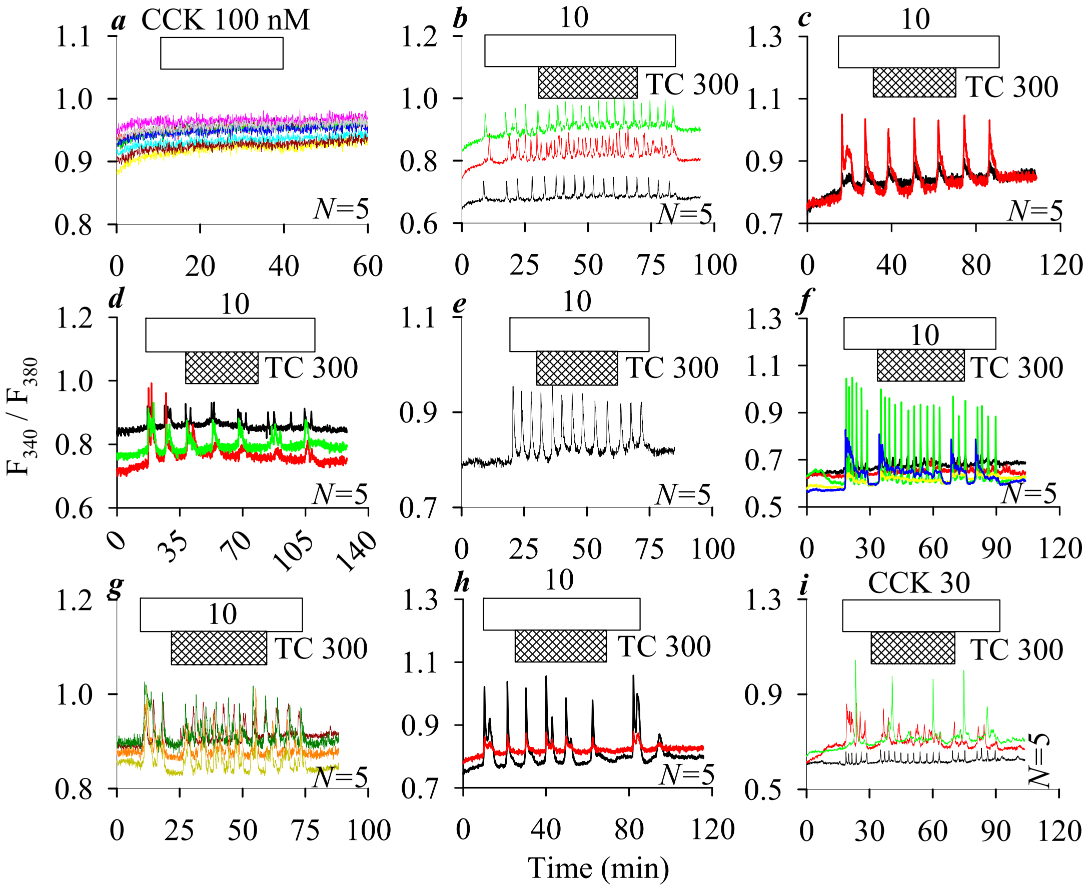

Figure S1. Trolox C had no effect on CCK-triggered calcium oscillations Parental CHO-K1 cells, and transfected CHO-K1 cells were loaed with Fura-2 AM and perifused, then CCK (empty bar), Trolox C (crossed bar) at indicated concentrations were added as indicated by the horizontal bars: (***a***) CHO-K1 cells, (***b-e***) CCK1R-CHO-K1, (***f-i***) CCK1R-miniSOG-CHO-K1 cells. (***a***) CCK 100 nM. (***b***) hCCK1R-CHO-K1, CCK 10 pM, Trolox C 300 μM. (***c***) rCCK1R-CHO-K1, CCK 10 pM, Trolox C 300 μM. (***d***) mCCK1R-CHO-K1, CCK 10 pM, Trolox C 300 μM. (***e***) pdCCK1R-CHO-K1, CCK 10 pM, Trolox C 300 μM. (***f***) hCCK1R-miniSOG-CHO-K1, CCK 10 pM, Trolox C 300 μM. (***g***) rCCK1R-miniSOG-CHO-K1, CCK 10 pM, Trolox C 300 μM. (***h***) mCCK1R-miniSOG-CHO-K1, CCK 10 pM, Trolox C 300 μM. (***i***) pCCK1R-miniSOG-CHO-K1, CCK 30 pM, Trolox C 300 μM. The original calcium traces shown in panels (***a-i***) are from one out of *N* identical experiments (*N* = 3-5), with each trace representing one individual cell.
